# Munificent Contribution to Medical Education

**Published:** 1848-04

**Authors:** 


### MUNIFICENT CONTRIBUTION TO MEDICAL EDUCATION.

Some twelve or fourteen years ago, a medical school was commenced under the name of Willoughby University, in the humble village of Chagrin, now Willoughby, eighteen miles from Cleveland, Ohio, on the banks of Lake Erie. A few years since, most of the Faculty left it for the more eligible city just named. Their places were supplied by others, who, however, soon felt the disadvantages of the position; and, with the sanction of their trustees and the Legislature of Ohio, they transferred the institution to Columbus, the seat of government. There it attracted the regard of LYNE STARLING, Esq., an old and respected citizen, formerly of Kentucky, who during the past winter made to it the liberal donation of THIRTY THOUSAND DOLLARS—two-thirds to be expended in the erection of a college edifice, and one-third in founding a hospital for clinical instruction. It gives us great pleasure to record this act of liberality, and we trust that all our readers will honor the name of Starling, as those to whom the money was given have done; for by an alteration in the charter, the school is henceforth to be entitled THE STARLING MEDICAL COLLEGE.

With the aid of this ample endowment, we trust the Faculty will venture to advance the price of their tickets, which is now only eight dollars each. We would not insist that in a region where the charges of physicians are lower than in many other parts of the country, they should be required, while studying, to pay the highest price for their tickets; but that just named is too low, and ought to be advanced to twelve dollars at least. Such an advance is due to the benefactor of the College.

The annual announcement, from which we have obtained the foregoing particulars, presents a catalogue of one hundred and thirty-eight pupils, of which twenty-five, or about eighteen per cent, are marked as practitioners. This number struck us as large, and taking up the Catalogue of the University of Louisville, we found that the whole number thus marked was, in four hundred and six, but sixteen, or a fraction less than four per cent. If a great number of the physicians of Ohio have gone into practice before graduating, it is cheering to find that so many have not lost sight of the duty of perfecting their university education. We hear much from the people about the great number annually graduated by our schools; but they ought rather to dread the still greater number who engage in practical duties without graduating—many, indeed, notwithstanding the great number of schools, without attending a single course of lectures in any.

